# Continualization of Probabilistic Programs With Correction

**DOI:** 10.1007/978-3-030-44914-8_14

**Published:** 2020-04-18

**Authors:** Jacob Laurel, Sasa Misailovic

**Affiliations:** ETH Zurich, Zurich, Switzerland; University of Illinois Urbana-Champaign, Department of Computer Science Urbana, Urbana, Illinois 61820 USA

**Keywords:** Probabilistic Programming, Program Transformation, Continuity, Parameter Synthesis, Program Approximation

## Abstract

Probabilistic Programming offers a concise way to represent stochastic models and perform automated statistical inference. However, many real-world models have discrete or hybrid discrete-continuous distributions, for which existing tools may suffer non-trivial limitations. Inference and parameter estimation can be exceedingly slow for these models because many inference algorithms compute results faster (or exclusively) when the distributions being inferred are continuous. To address this discrepancy, this paper presents Leios. Leios is the first approach for systematically approximating arbitrary probabilistic programs that have discrete, or hybrid discrete-continuous random variables. The approximate programs have all their variables fully continualized. We show that once we have the fully continuous approximate program, we can perform inference and parameter estimation faster by exploiting the existing support that many languages offer for continuous distributions. Furthermore, we show that the estimates obtained when performing inference and parameter estimation on the continuous approximation are still comparably close to both the true parameter values and the estimates obtained when performing inference on the original model.

### 1 Introduction

Probabilistic programming languages (PPLs) offer an intuitive way to model uncertainty by representing complex probability models as simple programs [28]. A probabilistic programming system then performs fully automated statistical inference on this program by conditioning on observed data, to obtain a posterior distribution, all while hiding the intricate details of this inference process.

Probabilistic inference is a computationally hard task, even for programs containing only Bernoulli distributions (#P-complete [18]), but prior work has shown that for many inference algorithms, continuous and smooth distributions (such as Gaussians) can be *significantly* easier to handle than the distributions having discrete components or discontinuities in their densities [15, 53, 52, 9, 56].

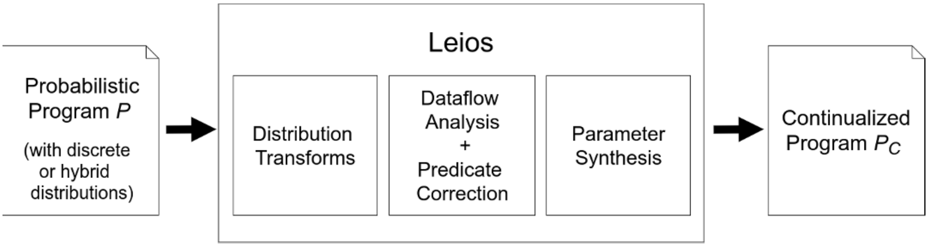

Fig. 1: Overview of Leios

However, many popular Bayesian models can have distributions which are discrete or hybrid discrete-continuous mixtures (denoted simply as "hybrid") leading to computationally inefficient inference for much the same reason. Particularly when the observed variable is a discrete-continuous mixture, inference may fail altogether [65]. Likewise even if the observed variable and likelihood are continuous, the prior or important latent variables, may be discrete (e.g., Binomial) leading to an equally difficult discrete inference problem [61, 50].

In fact, a number of popular inference algorithms such as Hamiltonian Monte Carlo [48], NUTS [31, 50], or versions of Variational Inference (VI) [9] only work for restricted classes of programs (e.g. by requiring each latent be continuous) to avoid these problems. Furthermore, we cannot always marginalize away the program's discrete component since it is often precisely the one we are interested in. Even if the parameter was one which could be safely marginalized out, doing so may require the programmer to use advanced domain knowledge to analytically solve and obtain a new model and re-write the program completely, which can be well beyond the abilities of the average PPL user.

**Problem statement:** We address the question of how to accurately approximate the semantics of a probabilistic program $P$ whose prior or likelihood is either discrete or hybrid, with a new program $P_C$, where all variables follow continuous distributions, so that we can exploit the aforementioned inference algorithms to improve inference in an easy, off-the-shelf fashion.

While a programmer could manually rewrite the probabilistic program or model and apply approximations in an ad hoc manner, such as simply adding Gaussian noise to each variable, this would be neither sufficient nor wise. For instance, it has been shown that when a model contains Gaussians, *how* they are programatically written and parametrized can impact the inference time and quality [29, 5]. Also, by not correcting for continuity in the program's branch conditions, one could significantly alter the probability of executing a particular program branch, and hence alter the overall distribution represented by the probabilistic program.

**Leios:** We introduce a fully automated program analysis framework to continualize probabilistic programs for significantly improved inference performance, especially in cases where inference was originally intractable or prohibitively slow.

An input to Leios is a probabilistic program, which consists of (1) *model* that specifies the prior distributions and how the latent variables are related,

(2) specifications of observable variables, and (3) specifications of data sets. Leios transforms the model, given the set of the observable variables. This model is then substituted back into the original program to produce a fully continuous probabilistic program leading to greatly improved inference. Furthermore the approximated program can easily be reused with different, unseen data.

Figure 1 presents the main workflow of Leios :

- *Distribution transformer and Boolean predicate correction:* Leios first finds individual discrete distribution sample statements to replace with continuous approximations based on known convergence theorems that specifically match the distributions' first moments [23]. Leios then performs a dataflow analysis to identify and then correct Boolean predicates in branches to best preserve the original program's probabilistic control flow. To correct Boolean predicates, we convert the program to a *sketch* and fill in the predicates with holes that will then be synthesized with the optimal values. We ensure that the distribution of the model's observed variables is fully continuous with a differentiable density function, by transforming it using an approach that adapts Smooth Interpretation [14] to probabilistic programs. We describe the transformations in Section 4.
- *Parameter Synthesizer:* Leios determines the optimal parameters which minimize a numerical approximation of the Wasserstein Distance to fill in the holes in the program sketch. This step of the algorithm can be thought of as a "training phase" much like in machine learning, and we need only perform it once for a given program, regardless of the number of times we will later perform inference on different data sets. These parameters correspond to *continuity correction factors* in classical probability theory [23]. We describe the synthesizer in Section 5.

**Contributions:** This paper makes the following main contributions:

- **Concept**: To the best of our knowledge, Leios is the first technique to automate program transformations that approximate discrete or hybrid discrete-continuous *probabilistic programs* with fully continuous ones to improve inference. It combines insights from probability theory, program analysis, compiler autotuning, and machine learning.
- **Program Transformation**: Leios implements a set of transformations on distributions and the conditional statements that can produce provably continuous probabilistic programs that approximate the original ones.
- **Parameter Synthesis**: We present a synthesis algorithm that corrects the probabilities of taking specific branches in the probabilistic program and improves the overall inference accuracy.
- **Evaluation**: We evaluated Leios on a set of ten benchmarks from existing literature and two systems, WebPPL (using MCMC sampling) and Pyro (using stochastic variational inference). The results demonstrate that Leios can achieve a substantial decrease in inference time compared to the original model, while still achieving high inference accuracy. We also show how a continualized program allows for easy off-the-shelf inference that is not always readily available to discrete or hybrid models.

```
1  Data := [12,8,...];
2
3  Model {
4   prior = Uniform(20,50);
5   Recruiters = Poisson(prior);
6
7   perfGPA = 4;
8   regGPA = 4*Beta(7,3);
9   GPA = Mix(perfGPA,.05,regGPA,.95)
10
11  if (GPA == 4) {
12   Interviews = Bin(Recruiters,.9);
13  } else if (GPA > 3.5) {
14   Interviews = Bin(Recruiters,.6);
15  } else {
16   Interviews = Bin(Recruiters,.5);
17  }
18
19  Offers = Bin(Interviews,0.4);
20 }
21
22 for d in Data {
23  factor(Offers,d);
24 }
25
26 return prior;
```

(a)

```
1  Model {
2   prior = Uniform(20,50);
3   mu_p = prior;
4   sigma_p = sqrt(prior);
5   Recruiters = Gaussian(mu_p,sigma_p);
6
7   perfGPA = Gaussian(4,β);
8   regGPA = 4*Beta(7,3);
9   GPA = Mix(perfGPA,.05,regGPA,.95)
10
11  if (4 - θ1 < GPA < 4+ θ2){
12   mu = Recruiters * 0.9;
13   sigma = sqrt(Recruiters*0.9*0.1);
14   Interviews = Gaussian(mu,sigma);
15  } else if (GPA > 3.5 + θ3){
16   mu = Recruiters * 0.6;
17   sigma= sqrt(Recruiters*0.6*0.4);
18   Interviews = Gaussian(mu,sigma);
19  } else {
20   mu = Recruiters * 0.5;
21   sigma = sqrt(Recruiters*0.5*0.5);
22   Interviews = Gaussian(mu,sigma);
23  }
24  mu2 = Interviews * 0.4;
25  sigma2 = sqrt(Interviews*0.4*0.6);
26  Offers = Gaussian(mu2,sigma2);
27 }
```

(b)

Fig. 2: (a) Program $P$ and (b) the Continualized Model Sketch

### 2 Example

Figure 2 (a) presents a program that infers the parameters of the distribution modeling the number of recruiters coming to a recruiting fair given both the number of offers multiple students receive (line 1). As the number of recruiters may vary year to year, we model this count as a Poisson distribution (line 5). However, to accurately quantify how *much* this count varies year to year, we want to estimate the unknown parameter of this Poisson variable. We thus place a uniform prior over this parameter (line 4).

The example represents the student GPAs in lines 7-9: it is either a perfect 4.0 score or any number between 0 and 4. We model the perfect GPA with a discrete distribution that has all the probability mass at 4.0 (line 7). To model the imperfect GPA, we use a Beta distribution (line 8), scaled by 4 to lie in the range $[0.0, 4.0]$. Finally, the distribution of the GPAs is a *mixture* of these two components (line 9). Our mixture assumes that 5% of students obtain perfect GPAs.

Because the GPA impacts the number of interviews a student receives, our model incorporates control flow where each branch captures the distribution of interviews received, conditioned on the GPA being in a certain range (lines 11-17). Each student's resume is available to all recruiters and each recruiter can request an interview or not, hence all three of the `Interviews` distributions follow a Binomial distribution (here denoted as `bin`) with the same $n$ (number of recruiters) but with different probabilities (higher probabilities for higher GPAs). From the factor statement (line 23) we see that the `Offers` variable governs the distribution of the observed data, hence it is the *observed* variable. Furthermore, given the values of all latent variables, `Offers` follows a Binomial distribution (line 19), hence the *likelihood function* of this program is discrete.

This program poses several challenges for inference. First, it contains discrete latent variables (such as the Binomials), which are expensive to sample from or rule out certain inference methods [26]. Second, it contains a hybrid discrete-continuous distribution governing the student GPA, and such hybrid distributions are challenging for inference algorithms [65]. Third, the model has complex control flow introduced by the `if` statements, making the observable data follow a (potentially multimodal) mixture distribution, which is yet another obstacle to efficient inference [43, 17]. Lastly, the discrete distribution of the observed data and likelihood also hinder the inference efficiency [61, 50, 59].

#### 2.1 Continualization

Our approach starts from the observation that inference with continuous distributions is often more efficient for several inference algorithms [53, 52, 56]. Leios first continualizes discrete and hybrid distributions in the original model. Starting in line 5 in Figure 2 (b), we approximate the Poisson variable with a Gaussian using a classical result [16], hence relaxing the constraint that the number of recruiters be an integer. (For ease of presentation we created new variables `mu_p` and `sigma_p` corresponding to the parameters of the approximation; Leios simply inlines these.) We next approximate the discrete component of the GPA hybrid mixture distribution by a Gaussian centered at 4 and small tunable standard deviation $\beta$ (line 7). The GPA is now a mixture of two *continuous* distributions. We then transform all of the Binomials to Gaussians (lines 14, 18, 22, and 26) using another classic approximation [23].

Finally, Leios smooths the observed variables by a Gaussian to ensure the likelihood function is both fully continuous *and* differentiable. In this example we see that the approximation of the Binomial already makes the distribution of `Offers` (given all latent values) a Gaussian, hence this final step is not needed.

After continualization, the GPA cannot be *exactly* 4.0, thus we need to repair the first conditional branch of the continualized program. In line 11, we replace the exact equality predicate with the interval predicate $\texttt{4-}\theta_1 \texttt{ < GPA < 4+}\theta_2$ where each $\theta$ is a hole whose value Leios will *synthesize*. Leios finds all such branching predicates by tracking transitive data dependencies of all continualized variables.

#### 2.2 Parameter Synthesis

Our continuous approximation should be close enough to the original model such that upon performing inference on the approximation, the estimations obtained will also be close to the ground-truth values. Hence Leios needs to ensure that the values synthesized for each $\theta$ are such that for every conditional statement, the probability of executing the true branch in the continualized program roughly matches the original (ensuring similar likelihoods). In probability theory, this value has a natural interpretation as a *continuity correction factor* as

```
1  Model {
2    prior = Uniform(20,50);
3    mu_p = prior;
4    sigma_p = sqrt(prior);
5    Recruiters = Gaussian(mu_p,sigma_p);
6
7    perfGPA = Gaussian(4, 0.1);
8    regGPA = 4*Beta(7,3);
9    GPA = Mix(perfGPA,.05,regGPA,.95);
10
11   if (3.99999 < GPA < 4.95208){
12     mu = Recruiters * 0.9;
13     sigma = sqrt(Recruiters*0.9*0.1);
14     Interviews = Gaussian(mu,sigma);
15   } else if (GPA > 3.500122){
16     mu = Recruiters * 0.6;
17     sigma = sqrt(Recruiters*0.6*0.4);
18     Interviews = Gaussian(mu,sigma);}
19   } else {
20     mu = Recruiters * 0.5;
21     sigma = sqrt(Recruiters*0.5*0.5);
22     Interviews = Gaussian(mu,sigma);
23   }
24
25   mu2 = Interviews * 0.4;
26   sigma2 = sqrt(Interviews*0.4*0.6);
27   Offers = Gaussian(mu2,sigma2);
28 }
```

(a)

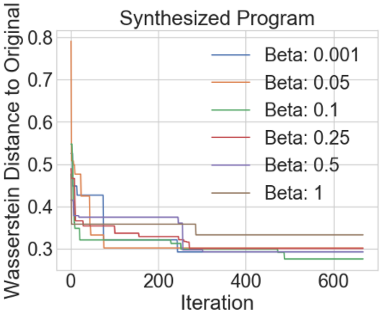

(b)

Fig. 3: (a) the fully continualized model and (b) Convergence of the Synthesis Step for multiple $\beta$.

it "corrects' the probability of a predicate being true after applying continuous approximations. For the `(GPA == 4)` condition, we might think about using a typical continuity correction factor of 0.5 [23], and transform it to `4-0.5 < GPA < 4+0.5`. However, in that case, the second `else if (GPA > 3.5)` branch would never execute, thus significantly changing the program's semantics (and thus the likelihood function). Experimentally, such an error can lead to highly inaccurate inference results.

Hence we must *synthesize* a better continuity correction factor that makes the approximated model "closest" to the original program's with respect to a well-defined distance metric between probability distributions. In this paper, we will use the common Wasserstein distance, which we describe later in Section 5. The objective function aims to find the continuity correction factors that minimize the Wasserstein distance between the original and continualized models.

Figure 3 (a) shows the continualized model. Leios calculated that the optimal values for the first branch are $\theta_1 = 0.00001$ (hence the lower bound is 3.99999) and $\theta_2 = 0.95208$ (hence the upper bound is 4.95208) in line 11, and $\theta_3 = 0.00012$ (hence the lower bound is 3.500122) for the branch in line 15. Intuitively the synthesizer found the upper bound 4.95208 so that any sample larger than 4 (which must have come from the right tail of the continualized perfect GPA) is consumed by the first branch, instead of accidentally being consumed by the second branch.

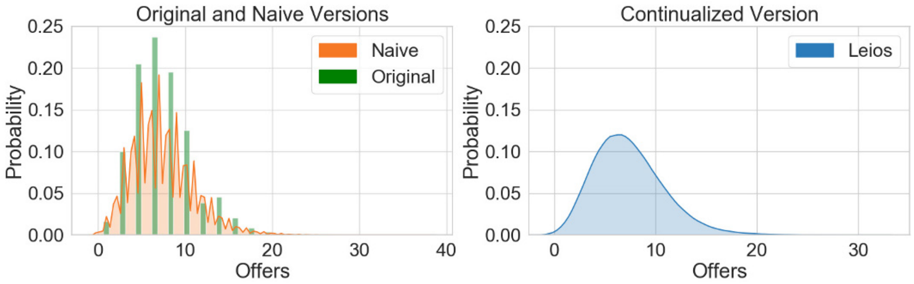

Fig. 4: Visual comparison between Model Distribution of Original Program with Naive Smoothing and Leios (both with $\beta = 0.1$)

Another part of the synthesis step is to make sure that approximations do not introduce run-time errors. Since `Interviews` is now sampled from Gaussian, there is a small possibility that it could become negative, thus causing a runtime error (since we later take its square root). By dynamically sampling the continualized model during the parameter synthesis, as part of a light-weight *auto-tuning* step, Leios checks if such an error exists. If it does, Leios can instead use a Gamma approximation (which is always non-negative).

While continualization incurs additional computational cost, this cost is typically amortized. In particular, continualization needs to be performed only once. The continualized model can be then be used multiple times for inference on different data-sets. Further, we experimentally observed that our synthesis step is fast. In this example, for all the values of $\beta$ we evaluated, this step required only a few hundred iterations to converge to the optimal continuity correction factors, as shown in Figure 3 (b).

#### 2.3 Improving Inference

Upon constructing the continuous approximation of the model, we now wish to perform inference by conditioning upon the outcomes of 25 sampled students. To make a fair comparison, we compile both the original and continuous versions down to Webppl [26] and run MCMC inference (with 3500 samples and a burn-in of 700). We also seek to understand how smoothing latent variables improves inference, thus we also compare against a naively continualized version *where only the observed variable* was smoothed using the same $\beta$, number of MCMC samples and burn-in.

Figure 4 presents the distribution of the `Offers` variable in the original model, naively smoothed model, and the Leios-optimized model. The continuous approximation achieved by Leios is smooth and unimodal, unlike the naively smoothed approximation, which is highly multimodal. However all models have similar means

Using these three models for inference, Figure 5 (a) presents the posterior distribution of the variable `param` for each approach. We finally take the *mean* as

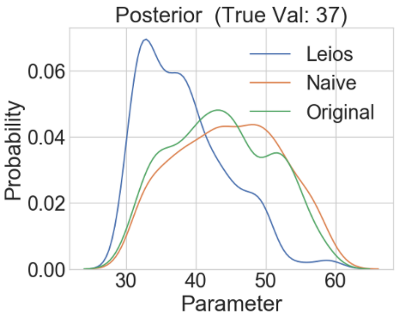

(a)

| Metric | Leios | Naive | Original |
|---|---|---|---|
| Accuracy | 0.058 | 0.069 | 0.090 |
| Runtime (s) | 0.604 | 0.631 | 0.805 |

(b)

Fig. 5: (a) *Posteriors* of each method – the true value is equal to 37. (b) Avg. Accuracy and Inference time; the bars represent accuracy (left Y-axis), the lines represent time (right Y-axis).

the point-estimate, $\tau_{est}$, of the parameter's true value $\tau$. Figure 5 (b) presents the run time and the error ratio, $|\frac{\tau-\tau_{est}}{\tau}|$, for each approach (for the given true value of 37). It shows that our continualized version leads to the fastest inference.

## 3 Syntax and Semantics of Programs

We present the syntax and semantics of the probabilistic programming language on which our analyses is defined

### 3.1 Source Language Syntax

$$
\begin{array}{lll}
\mathit{Program} & ::= & \mathit{DataBlock}?\ ;\ \mathit{Model}\ \{\ \mathit{Stmt}^{+}\ \}\ ;\ \mathit{ObserveBlock}?;\ \mathit{return}\ \ \mathit{Var}; \\
\mathit{Stmt} & ::= & \mathit{skip} \mid \mathit{abort} \mid \mathit{Var} := \mathit{Expr} \mid \mathit{Var} := \mathit{Dist} \mid \texttt{CONST}\ \mathit{Var} := \mathit{Expr} \\
 & \mid & \mathit{Stmt}\ ;\ \mathit{Stmt} \mid \{\ \mathit{Stmt}\ \} \mid \mathit{condition}\ (\ \mathit{BExpr}\ ) \\
 & \mid & \mathit{if}\ (\ \mathit{BExpr}\ )\ \mathit{Stmt}\ \mathit{else}\ \mathit{Stmt} \mid \mathit{for}\ i = \mathit{Int}\ \mathit{to}\ \mathit{Int}\ \mathit{Stmt} \\
 & \mid & \mathit{while}\ (\ \mathit{BExpr}\ )\ \mathit{Stmt} \\
\mathit{Expr} & ::= & \mathit{Expr}\ \mathit{ArithOp}\ \mathit{Expr} \mid f(\mathit{Expr}) \mid \mathit{Real} \mid \mathit{Int} \mid \mathit{Var} \\
\mathit{BExpr} & ::= & \mathit{BExpr}\ \mathit{or}\ \mathit{BExpr} \mid \mathit{BExpr}\ \mathit{and}\ \mathit{BExpr} \mid \mathit{not}\ \mathit{BExpr} \\
 & \mid & \mathit{Expr}\ \mathit{RelOp}\ \mathit{Expr} \mid (\ \mathit{BExpr}\ ) \\
\mathit{DataBlock} & ::= & \mathit{Data}{:=}\ [(\mathit{Int} \mid \mathit{Real})^{*}] \\
\mathit{ObserveBlock} & ::= & \mathit{for}\ D\ \mathit{in}\ \mathit{Data}\ \{\ \mathit{factor(Var,D);}\ \} \\
\mathit{Dist} & ::= & \mathit{ContDist} \mid \mathit{DiscDist}
\end{array}
$$

$$\mathit{ContDist} \in \{\mathit{Gaussian}, \mathit{Uniform}, \mathit{etc.}\}, \mathit{DiscDist} \in \{\mathit{Binomial}, \mathit{Bernoulli}, \mathit{etc.}\}$$

$$\mathit{ArithOp} \ \in \{+, -, *, /, **\},\ \ f \in \{\mathit{log}, \mathit{abs}, \mathit{sqrt}, \mathit{exp}\},\ \ \mathit{RelOp} \in \{<, \leq, ==\}$$

The syntax is similar to the ones used in [24, 51]. Unlike [51], our syntax *does* include exact equality predicates, which introduce difficulties during the approximation. To give the developer the flexibility in selecting which parts of the program to continualize, we add the `CONST` annotation. It indicates that the variable's distribution should not be continualized. Until explicitly noted, we will not use this annotation in the rest of the paper. For simplicity of exposition, we present only a single *DataBlock* and *ObserveBlock*, but our approach naturally extends to the cases with multiple data and observed variables.

**Measure Theory Preliminaries** Though various semantics have been proposed [44, 36, 7], we adapt the sub-probability measure transformer semantics of Dahlqvist et al. [19]. We will use the terms distribution and measure interchangeably.

**Definition 1.** *A program state $\sigma \in \mathbb{S}$ is a n-tuple of real numbers: $\mathbb{S} = \mathbb{R}^n$ where the $i^{th}$ tuple element corresponds to the $i^{th}$ program variable's value.*

**Definition 2.** *A $\Sigma$-algebra on a set $X$ (denoted as $\Sigma_X$) is a collection of subsets of $X$ such that (1) $X \in \Sigma_X$ and (2) $X_i \in \Sigma_X \Rightarrow X_i^c \in \Sigma_X$ (closure under complementation) and (3) $X_1, X_2 \in \Sigma_X \Rightarrow X_1 \vee X_2 \in \Sigma_X$ (closure under countable union). The tuple of $(X, \Sigma_X)$ is called a measurable space. Our semantics is defined on the Borel measurable space $(\mathbb{R}^n, \mathcal{B}\{\mathbb{R}^n\})$ where $\mathcal{B}\{\mathbb{R}^n\}$ is the standard Borel $\Sigma$-algebra over $\mathbb{R}^n$.*

**Definition 3.** *A measure $\mu$ over $\mathbb{R}^n$ is a mapping from $\mathcal{B}\{\mathbb{R}^n\}$ to $[0, +\infty)$ such that $\mu(\emptyset) = 0$ and $\mu(\bigcup_{i\in\mathbb{N}} X_i) = \sum_{i\in\mathbb{N}} \mu(X_i)$ when all $X_i$ are mutually disjoint. A probability measure is a measure that satisfies $\mu(\mathbb{R}^n) = 1$ and a sub-probability measure is one satisfying $\mu(\mathbb{R}^n) \leq 1$. The simplest measure is the Dirac measure denoted as $\delta_{a_i}(S) =$ 1 if $a_i$ in $S$ else 0. We denote the set of all sub-probability measures as $\mathbb{M}(\mathbb{R}^n)$.*

**Definition 4.** *Given measures $\mu_1, \mu_2 \in \mathbb{M}(\mathbb{R})$, the product measure $\mu_1 \otimes \mu_2 \in \mathbb{M}(\mathbb{R}^2)$ is defined as $\mu_1 \otimes \mu_2(B_1 \times B_2) = \mu_1(B_1)\mu_2(B_2)$ for $B_1, B_2 \in \mathcal{B}\{\mathbb{R}\}$*

**Definition 5.** *Given a measure $\mu \in \mathbb{M}(\mathbb{R}^n)$ the marginal measure of a variable $x_i$ is defined as $\mu_{x_i}(B_i) = \mu(\mathbb{R} \times ...\mathbb{R} \times B_i \times \mathbb{R}...)$ for $B_i \in \mathcal{B}\{\mathbb{R}\}$*

**Definition 6.** *A kernel is a function $\kappa : \mathbb{S} \to \mathbb{M}(\mathbb{R}^n)$ mapping states to measures.*

**Definition 7.** *The Lebesgue measure on $\mathbb{R}$ (denoted Leb) is the measure that maps any interval to its length, e.g., $Leb([a, b]) = b - a$. The Lebesgue measure in $\mathbb{R}^n$ is simply the n-fold product measure of $n$ copies of the Lebesgue measure on $\mathbb{R}$.*

**Definition 8.** *A measure $\mu$ is absolutely continuous with respect to the Lebesgue measure Leb (denoted as $\mu \ll Leb$ or simply $\mu$ is A.C.) iff for any measurable set $S$ $Leb(S) = 0 \Rightarrow \mu(S) = 0$.*

#### 3.2 Semantics

**Expression Level Semantics** Arithmetic Expression semantics are standard, they map states $\sigma \in \mathbb{R}^n$ to values, equivalently $[\![Expr]\!] : \mathbb{R}^n \to \mathbb{R}$. Boolean Expression Semantics, denoted $[\![BExpr]\!]$, simply return the set of states $B_i \in \mathcal{B}\{\mathbb{R}^n\}$ satisfying the Boolean conditional.

$$[\![c]\!](\sigma) = c \quad [\![x_i]\!](\sigma) = \sigma[x_i] \quad [\![t_1\ op\ t_2]\!](\sigma) = [\![t_1]\!](\sigma)\ op\ [\![t_2]\!](\sigma) \quad [\![f(t_1)]\!](\sigma) = f([\![t_1]\!](\sigma))$$

$$[\![B_1\ \texttt{and}\ B_2]\!] = [\![B_1]\!] \cap [\![B_2]\!] \qquad [\![B_1\ \texttt{or}\ B_2]\!] = [\![B_1]\!] \cup [\![B_2]\!] \qquad [\![\texttt{not}\ B_1]\!] = \mathbb{R}^n \setminus [\![B_1]\!]$$

$$[\![e_1\ relop\ e_2]\!] = \{\sigma \in \mathbb{R}^n \mid [\![e_1]\!](\sigma)\ relop\ [\![e_2]\!](\sigma)\}$$

**Distribution Semantics** The interpretation of a distribution is a kernel, $\kappa$, mapping a state to the measure associated with the specific parametrization of the distribution in that state. Since measures are set functions we will represent them as $\lambda$ abstractions. The signature is $[\![Dist]\!] : \mathbb{R}^n \to (\mathcal{B}\{\mathbb{R}\} \to [0,1])$

$$\kappa_{Cont}(\sigma) = [\![ContDist(e_1, e_2, ...)]\!](\sigma) = \lambda S. \int_{v \in \mathbb{R}} \mathbf{1}_S(v) \cdot f_{Cont}(v; [\![e_1]\!](\sigma), [\![e_2]\!](\sigma), ...)$$

$$\kappa_{Disc}(\sigma) = [\![DiscDist(e_1, e_2, ...)]\!](\sigma) = \lambda S. \sum_{v \in Supp \cap S} f_{Disc}(v; [\![e_1]\!](\sigma), [\![e_2]\!](\sigma), ...)$$

Where $f_{Cont}$ and $f_{Disc}$ are the density and mass functions, respectively, of the primitive distribution being sampled from (e.g., $f_{Gauss}(x; \mu, \sigma) = \frac{1}{\sigma\sqrt{2\pi}} e^{\frac{-(x-\mu)^2}{2\sigma^2}} \cdot \mathbf{1}_{\{\sigma > 0\}}$) and *Supp* is the distribution's support.

**Statement Level Semantics** The statement-level semantics are shown in Figure 6. We interpret each statement as a (sub) measure transformer, hence the semantic signature is $[\![Statement]\!] : \mathbb{M}(\mathbb{R}^n) \to \mathbb{M}(\mathbb{R}^n)$ . The `skip` statement returns the original measure and the `abort` statement transforms any measure to the **0** sub-measure. The `condition` statement removes measure from regions not satisfying the Boolean guard $B$. The `factor` statement can be seen as a "smoothed" version of condition that uses $g$, a function of the observed data and its distribution, to re-weight the measure associated with a set by some real value in $[0, 1]$ (as opposed to strictly 0 or 1). Deterministic assignment transforms the measure into one which assigns to any set of states $S$ the same value that the original measure $\mu$ would have assigned to all states that end up in $S$ after executing the assignment statement. Probabilistic Assignment updates the measure so that $x_i$'s marginal is the measure associated with `Dist`, but with the parameters governed by $\mu$.

An `if else` statement can be decomposed into the sum of the true branch's measure and the false branch's measure. The `while` loop semantics are the solution to the standard least fixed point equation [19], but can also be viewed as a mixture distribution where each mixture component corresponds to going through the loop $k$ times. A `for` loop is just syntactic sugar for a sequencing of a fixed number of statements. We note that the Data block does not affect the measure (it is also syntactic sugar, and could simply be inlined in the Observe block). The program can be thought of as starting in some initial input measure $\mu_0$ where each variable is undefined (which could simply mean initialized to some special value or even just zero), and as each variable gets defined, that variable's marginal (and hence the joint measure $\mu$) gets updated.

## 4 Continualizing Probabilistic Programs

Our goal is to synthesize a new continuous approximation of the original program $P$. We formally define this via a transformation operator $\mathcal{T}_{\mathcal{P}}^{\beta}[\bullet]$: *Program* $\to$ *Program*. Our approach operates in two main steps:

(1) We first *locally* approximate the program's prior and latent variables using a series of program transformations to best preserve the local structural properties of the program and then apply smoothing *globally* to ensure that the likelihood function is both fully continuous and differentiable.

$$[\![\texttt{skip}]\!](\mu) = \mu \qquad [\![\texttt{abort}]\!](\mu) = \lambda S.0 \qquad [\![P_1; P_2]\!](\mu) = [\![P_2]\!]([\![P_1]\!](\mu))$$

$$[\![\texttt{condition(B)}]\!](\mu) = \lambda S.\mu(S \cap [\![B]\!]) \qquad [\![\texttt{factor(x}_i\texttt{,t)}]\!](\mu) = \lambda S.\int_{\mathbb{R}^n} \mathbf{1}_S \cdot g(t,\sigma)\cdot \mu(d\sigma)$$

$$[\![\texttt{x}_i \texttt{ := e}]\!](\mu) = \lambda S.\mu(\{(x_1,...,x_n) \in \mathbb{R}^n \mid (x_1,...,x_{i-1},[\![e]\!](x_1,..x_n),x_{i+1}...,x_n) \in S\})$$

$$[\![\texttt{x}_i \texttt{ := Dist(e}_1\texttt{,...e}_k\texttt{)}]\!](\mu) = \lambda S.\int_{\mathbb{R}^n} \mu(d\sigma)\cdot\delta_{x_1}\otimes...\delta_{x_{i-1}}\otimes[\![\texttt{Dist(e}_1\texttt{,...e}_k\texttt{)}]\!](\sigma)\otimes\delta_{x_{i+1}}...(S)$$

$$[\![\texttt{if (B) }\{P_1\}\texttt{ else }\{P_2\}]\!](\mu) = [\![P_1]\!]([\![\texttt{condition(B)}]\!](\mu)) + [\![P_2]\!]([\![\texttt{condition(not B)}]\!](\mu))$$

$$[\![\texttt{while (B) \{ P}_1\texttt{ \}}]\!](\mu) = \sum_{k=0}^{\infty} [\![(\texttt{condition(B); P}_1)^k; \texttt{condition(not B)}]\!](\mu)$$

Fig. 6: Denotational Semantics of Probabilistic Programs

(2) We next synthesize a set of parameters that (approximately) minimize the distance metric between the distributions of the original and continualized models and we use light-weight *auto-tuning* to ensure the approximations do not introduce runtime errors.

#### 4.1 Overview of the Algorithm

Algorithm 1 presents the technique for continualizing programs. It takes as input a program $P$ containing a prior or observed variable that is discrete (or hybrid) and returns $\mathcal{T}_{\mathcal{P}}^{\beta}[P]$, a probabilistic program representing a fully continuous random variable with a differentiable likelihood function. The algorithm uses a tunable hyper-parameter $\beta \in (0,\infty)$ to control the amount of smoothing (like in [14]). A smaller $\beta$ leads to less smoothing, while a larger $\beta$ leads to more smoothing, however the smallest $\beta$ does not always lead to the best inference, and vice-versa, as can be seen in section 7.

In line 3 of Algorithm 1 Leios constructs a standard control flow graph (CFG) to represent the program, using a method called `GetCFG()`. This data structure will form the basis of Leios's future analyses. Each CFG node corresponds to a single statement and contains all relevant attributes of that statement. Leios then uses this CFG to build a data dependency graph (line 4) which will be used for checking which variables are tainted by the approximations. In line 5 Leios then applies $\mathcal{T}_{\mathcal{P}}^{\beta}[\bullet]$ to obtain a continualized sketch, $P_C$. Lastly, Leios synthesizes the optimal continuity correction parameters (line 7), and in doing so, samples the program to detect if a runtime error occurred, also returning a Boolean flag *success* to convey this information. If a runtime error *did* occur we find the expression causing it (line 9) and then in lines 10-12 reapply the safer transformations (e.g., Gamma instead of Gaussian) to all possible dependencies which could have contributed to the runtime error.

#### 4.2 Distribution and Expression Transformations

To continualize each variable, Leios mutates the individual distributions and expressions assigned to latent variables within the program. We use a transform operator for expressions and distributions $\mathcal{T}_{\mathcal{E}}^{\beta}[\bullet]$: $Expr \cup Dist \rightarrow Expr \cup Dist$, which we define next.

**Algorithm 1:** Procedure for Continualizing a Probabilistic Program function Continualize $(P, \beta)$;
**Input :** A probabilistic program $P$ containing discrete/hybrid observable variables and/or priors and a smoothing factor $\beta > 0$
**Output:** A fully continuous probabilistic program $P_C$
$Acceptable \leftarrow False$;
$CFG \leftarrow$ `GetCFG`$(P)$;
$DataDepGraph \leftarrow$ `ComputeDataFlow`$(CFG)$;
$P_C \leftarrow \mathcal{T}_{\mathcal{P}}^{\beta}[P]$; `/* apply all continuous transformations */`
**while** ***not*** *Acceptable* **do**
  $P_C, success \leftarrow$ `Synthesize`$(P_C, P)$;
  **if** ***not*** *success*:
    $D \leftarrow$ `getInvalidExpression()`;
    $Deps \leftarrow$ `getDependencies(DataDepGraph,D)`;
    **forall** *Expression* **in** *Deps* **do**
      $P_C \leftarrow$ `reapplySafeTransformation`$(P_C,$ *Expression*$)$;
  **else**:
    $Acceptable \leftarrow True$;
**end**
**return** $P_C$

**Transform Operator For Distributions and Expressions** We now detail the full list of continuous probability distribution transformations that $\mathcal{T}_{\mathcal{E}}^{\beta}[\bullet]$ uses.

$$\mathcal{T}_{\mathcal{E}}^{\beta}[E] = \begin{cases} Gaussian(\lambda, \sqrt{\lambda}) & E = Poisson(\lambda) \\ Gamma(\lambda, 1) & E = Poisson(\lambda) \text{ \& } Gaussian\ fails \\ Gaussian(np, \sqrt{np(1-p)}) & E = Binomial(n, p) \\ Gamma(n, p) & E = Binomial(n, p) \text{ \& } Gaussian\ fails \\ Uniform(a, b) & E = DiscUniform(a, b) \\ Exponential(p) & E = Geometric(p) \\ MixOfGauss_{\beta}([(1, p), (0, 1-p)]) & E = Bernoulli(p) \\ Beta(\beta, \beta\frac{1-p}{p}) & E = Bernoulli(p) \text{ \& } MixOfGauss\ fails \\ Mixture([(\mathcal{T}_{\mathcal{E}}^{\beta}[D_1], p_1), ...(\mathcal{T}_{\mathcal{E}}^{\beta}[D_2], p_2)]) & E = Mixture([(D_1, p_1), ...(D_2, p_2)]) \\ Gaussian(c, \beta) & E = c\ (constant) \\ E & E = a{\cdot}x_i{+}b\ (a{\neq}0) \\ KDE(\beta) & E \in DiscDist \text{ \& } not\ covered \\ Gaussian(E, \beta) & otherwise \end{cases}$$

The rationale for this definition is that these approximations all preserve key structural properties of the distributions' shape (e.g., the number of modes) which have been shown to strongly affect the quality of inference [25, 45, 17]. Second, these continuous approximations all match the first moment of their corresponding discrete distributions, which is another important feature that affects the quality of approximation [53]. We refer the reader to [54] to see that for each distribution on the left, the corresponding continuous distribution on the right has the same mean. These approximations are best when certain limit conditions are satisfied, e.g. $\lambda \geq 10$ for approximating a Poisson distribution with Gaussian, hence the values in the program itself do affect the overall approximation accuracy.

However, if we are not careful, a statement level transformation could introduce runtime errors. For example, a Binomial is always non-negative, but its Gaussian approximation could be negative. This is why $\mathcal{T}_{\mathcal{E}}^{\beta}[\bullet]$ has *multiple* transformations for the same distribution. For example, in addition to using a Gaussian to approximate both a Binomial and a Poisson, we also have a Gamma approximation since a Gamma distribution is *always non-negative.* Likewise we have a Beta approximation to a Bernoulli if we require that the approximation also have support in the range $[0, 1]$. Leios uses auto-tuning to safeguard against such errors during the synthesis phase, whereby when sampling the transformed program, if we encounter a run-time error of this nature, we simply go back and try a safer (but possibly slower) alternative (Algorithm 1 line 12). Since there are only finitely many variables and (safer) transformations to apply, this process will eventually terminate. For discrete distributions *not* supported by the specific approximations, but with fixed parameters, we empirically sample them to get a set of samples and then use a Kernel Density Estimate (KDE) [62] with a Gaussian kernel (the KDE bandwidth is precisely $\beta$) as the approximation.

Lastly, by default *all* discrete random variables become approximated with continuous versions, however we leave the option to the user to manually specify `CONST` in front of a variable if they *do not* wish for it to be approximated (in which case we no longer make any theoretical guarantees about continuity).

#### 4.3 Influence Analysis and Control-Flow Correction of Predicates

Simply changing all instances of discrete distributions in the program to continuous ones is not enough to closely approximate the semantics of the original program. We additionally need to ensure that such changes do not introduce *control flow errors* into the program, in the sense that quantitative properties such as the probability of taking a particular branch need to be reasonably preserved.

**Avoiding Zero Probability Events** A major concern of the approximation is to ensure that no zero-probability events are introduced, such as when we have an exact equality "`==`" predicate in an `if`, `observe` or `while` statement and the variable being checked was transformed from a discrete to a continuous type. For example, discrete programs commonly have a statement like `x := Poisson(1)` followed by a conditional such as `if (x==4)`, because the probability that a discrete random variable is *exactly* equal to a value can be non-zero. However upon applying our distribution transformations and transforming the distribution of $x$ from a discrete Poisson to a continuous Gaussian, the conditional statement "`if (x==4)`" now corresponds to a **zero probability** (or measure zero) event, as the probability that an absolutely continuous probability measure assigns to the singleton set $\{4\}$ is by definition zero. Thus, if not corrected for, we could significantly change the probabilities of taking certain branches and hence the overall distribution of the program.

The converse can also be true: applying approximations can make a zero probability event in the original program now have non-zero probability. For example, in `x := DiscUniform(1,5); if (x<3 and x>2)` the true branch has probability zero of executing but this becomes non-zero after approximations are applied. However, the branch paths like these in the original model could be identified by symbolic analysis (e.g., [24]) and removed via dead code elimination during pre-processing.

**Correcting Control Flow Probabilities via Static Analysis** To prevent zero-probability events and ensure that the branch execution probabilities of the continualized program closely matches the original's, we use data dependence analysis to track which `if`, `while` or `condition` statements have logical comparisons with variables "tainted" by the approximations. A variable $v$ is "tainted" if it has a transitive data dependence on an approximated variable, and we use *reaching definitions analysis* [35] on the program's CFG to identify these.

As shown in Algorithm 1 line 4, to compute the reaching definitions analysis we use a method called `ComputeDataFlow()` as part of a pre-transformation pass whereby for each program point in the CFG, each variable is marked with all the other variables on which it has a data-dependence. These annotations are stored in a data structure called *DataDepGraph* which maps *nodes* (program points) to *sets of tuples* where each tuple contains a variable, the other variables it depends on (and where they are assigned), and lastly, whether it will become tainted. Note that in the algorithm this step is done *before* the previously discussed expression-level transformations, hence why `ComputeDataFlow()` marks which variables will *become* continualized and which ones will not (i.e if a variable already defines a continuous random variable or was annotated with `CONST`). Furthermore, though we are computing the data dependencies before the approximations, because the approximations do not re-order or remove statements, all data dependencies will be the same before and after applying the approximations.

**Transform Operator For Boolean Expressions** We take all such control predicates that contain an exact equality "`==`" comparison with a tainted variable and transform these predicates from exact equality predicates to interval-style predicates. Thus if we originally had a predicate of the form `if(x==4)` we will mutate this into a predicate of the form `if(x>4-`$\theta_1$ `&& x<4+`$\theta_2$`)` where $\theta$ are now placeholder values that will need to be filled with a concrete value during the synthesis phase (Section 5). Hence checking for exact equality gets relaxed to checking for containment within the interval $(4-\theta_1, 4+\theta_2)$. We also need to correct `<` and `<=` predicates if one of the variables was approximated or transitively affected by an approximation.

Hence we also define our transform operator $\mathcal{T}_{\mathcal{B}}^{\beta}[\bullet] : BExpr \rightarrow BExpr$ at the level of Boolean expressions:

$$\mathcal{T}_{\mathcal{B}}^{\beta}[(x == y)] = \begin{cases} (y - \theta_1 < x) \ \textit{and} \ (x < y + \theta_2) & \textit{default} \\ (x == y) & \texttt{CONST } \texttt{x} \textit{ and } \texttt{CONST } \texttt{y} \textit{ specified} \end{cases}$$

$$\mathcal{T}_{\mathcal{B}}^{\beta}[(x < y)] = \begin{cases} (x < y + \theta) & \textit{if } x \textit{ or } y \textit{ tainted} \\ (x < y) & \textit{otherwise} \end{cases}$$

$$\mathcal{T}_{\mathcal{B}}^{\beta}[(x \leq y)] = \begin{cases} (x \leq y + \theta) & \textit{if } x \textit{ or } y \textit{ tainted} \\ (x \leq y) & \textit{otherwise} \end{cases}$$

Because we have already pre-computed *DataDepGraph* one can check if a variable in a given statement or expression is tainted (or marked as `CONST`) in constant time.

This correction has a natural interpretation in classical probability theory. It is well known that to approximate a discrete distribution $X$ with a continuous one $\hat{X}$, we need a continuity correction factor, $\theta$, such that $P(X < x) \approx P(\hat{X} < x + \theta)$ (hence why $\mathcal{T}_{\mathcal{B}}^{\beta}[\bullet]$ also corrects `<` and `<=` predicates). For simple approximations (i.e Binomial to Gaussian), the canonical correction factor is known ($\theta = 0.5$) [23], however for the general case, it is not. Furthermore, it has been shown that in many cases, 0.5 is *not* the best correction factor [3].

#### 4.4 Bringing it all together: Full Program Transformations

Having defined the transformation for distributions, arithmetic and Boolean expressions, we now define the *program* transformation operator $\mathcal{T}_{\mathcal{P}}^{\beta}[\bullet]$: $Program \rightarrow Program$ inductively:

$$\begin{aligned}
\mathcal{T}_{\mathcal{P}}^{\beta}[P_1; P_2] &= \mathcal{T}_{\mathcal{P}}^{\beta}[P_1]; \mathcal{T}_{\mathcal{P}}^{\beta}[P_2] \\
\mathcal{T}_{\mathcal{P}}^{\beta}[\texttt{if (B) } \{P_1\} \texttt{ else } \{P_2\}] &= \texttt{if (}\mathcal{T}_{\mathcal{B}}^{\beta}[B]\texttt{) } \mathcal{T}_{\mathcal{P}}^{\beta}[P_1] \texttt{ else } \mathcal{T}_{\mathcal{P}}^{\beta}[P_2] \\
\mathcal{T}_{\mathcal{P}}^{\beta}[\texttt{while(B) } P_1] &= \texttt{while(}\mathcal{T}_{\mathcal{B}}^{\beta}[B]\texttt{) } \mathcal{T}_{\mathcal{P}}^{\beta}[P_1] \\
\mathcal{T}_{\mathcal{P}}^{\beta}[\texttt{condition(B)}] &= \texttt{condition(}\mathcal{T}_{\mathcal{B}}^{\beta}[B]\texttt{)} \\
\mathcal{T}_{\mathcal{P}}^{\beta}[\texttt{x := E}] &= \texttt{x := } \mathcal{T}_{\mathcal{E}}^{\beta}[E] \\
\mathcal{T}_{\mathcal{P}}^{\beta}[\texttt{CONST x := E}] &= \texttt{x := E}
\end{aligned}$$

The `abort`, `factor` and `skip` statements and the *DataBlock* remain the same after applying the transformation operator $\mathcal{T}_{\mathcal{P}}^{\beta}[\bullet]$.

**Ensuring Smoothness** Upon applying the statement-level transformations and performing both dataflow analysis and predicate mutations, Leios ensures each latent variable comes from a continuous distribution. However a continuous distribution may still have jump discontinuities or non-differentiable regions in its density function (such as a uniform distribution), which can make inference difficult [66]. Furthermore it is known that performing parameter estimation on data that is distributed according to a discontinuous or non-smooth density function, or on distributions with a non-smooth likelihoods can be just as challenging [50, 1, 59]. Thus to make the Program's likelihood function and density function of the observed data fully *smooth*, we need to apply additional Gaussian smoothing.

Since it would be redundant to apply smoothing if we already knew this variable came from a smooth distribution (as in the example) hence we make this simple check first. The following transformation performs this on the observed variables (which appear in the factor statement).

$$\mathcal{T}_{\mathcal{P}}^{\beta}[\texttt{x}_o \texttt{ := E}] = \begin{cases} \texttt{x}_o \texttt{ := E} & \textit{if x already smooth} \\ \texttt{x}_o \texttt{ := Gaussian(E,}\beta\texttt{);} & \textit{otherwise} \end{cases}$$

We could perform additional smoothing for *every* variable to ensure each has a differentiable density, however we empirically observed that the variance added up enough to where inference quality deteriorated, hence we only apply the additional smoothing to observed variables.

Having defined the statement-level transformations we now state a theorem about $\mathcal{T}_{\mathcal{P}}^{\beta}[\bullet]$ preserving continuity. As many applications may invoke inference at any point in the program [46, 60], it is important that absolute continuity of each marginal hold at *every* point.

**Theorem 1.** *In the transformed program, $\mathcal{T}_{\mathcal{P}}^{\beta}[P]$, the marginal sub-probability measure of each variable, denoted $\mu_{x_i}$, is absolutely continuous with respect to the Lebesgue measure (denoted $\mu_{x_i}$ is A.C.) at each program point for which that variable is defined.*

*Proof.* (sketch) To prove the theorem we will show that when any variable $x_i$ is initially defined, it comes from an absolutely continuous distribution and furthermore that the semantics of each statement in $\mathcal{T}_{\mathcal{P}}^{\beta}[P]$ preserves the absolute continuity of each marginal measure (where $\mu_{x_i} \equiv \mu(\mathbb{R} \times ... \times B_i \times \mathbb{R} ... \times \mathbb{R})$), equivalently for any statement, any (already defined) variable $x_i$ and any Borel set $B_i \in \mathcal{B}\{\mathbb{R}\}$:

$$\mu(\mathbb{R} \times ... \times B_i \times \mathbb{R} ... \times \mathbb{R}) \text{ is A.C.} \Rightarrow [\![statement]\!](\mu)(\mathbb{R} \times ... \times B_i \times \mathbb{R} ... \times \mathbb{R}) \text{ is A.C.}$$

*Case 1.* `skip` and `abort`: Since `skip` is the identity measure transformer of each defined marginal measure $\mu_{x_i}$ was A.C. before, then they will trivially be so afterward since they are unchanged. `abort` sends each marginal to the **0** sub-measure (which is trivially A.C.).

*Case 2.* `condition` and `factor`: Since `factor` and `condition` only lose measure we have $[\![\texttt{condition(B)}]\!](\mu)(S) \leq \mu(S)$ and $[\![\texttt{factor(x}_k\texttt{,t)}]\!](\mu)(S) \leq \mu(S)$ for any Borel set $S$. Thus $\mu(S) = 0 \Rightarrow [\![\texttt{condition(B)}]\!](\mu)(S) = 0$ and $\mu(S) = 0 \Rightarrow [\![\texttt{factor(x}_k\texttt{,t)}]\!](\mu)(S) = 0$ since all measures are non-negative. Hence by transitivity, since $\mu(\mathbb{R} \times ... B_i \times \mathbb{R} ...)$ *is A.C.*, $[\![\texttt{factor(x}_k\texttt{,t)}]\!](\mu)(S)(\mathbb{R} \times ... B_i \times \mathbb{R} ... \times \mathbb{R})$ *is A.C.* and likewise for similar reasons, we have that $[\![\texttt{condition(B)}]\!](\mu)(\mathbb{R} \times ... B_i \times \mathbb{R} ... \times \mathbb{R})$ *is A.C.*

*Case 3.* Assignment: Probabilistic assignment is straightforward. Since the continualized program only samples from absolutely continuous distributions, the marginal of the sampled variable $x_i$ will be A.C. and all other marginals $\mu_{x_j}$ were A.C. by assumption. Deterministic assignment has to be handled carefully. In the continualized program the only deterministic assignments will be $\texttt{x}_i$ `:= a*`$x_j$`+b;` for $a \neq 0$ (all other assignments are smoothed). The marginal $\mu_{x_i}(S)$ is just $\mu_{x_j}(aS + b)$ where the set $aS + b \equiv \{s \in \mathbb{R} \mid a \cdot s + b \in S\}$. However by assumption of the A.C. of $x_j$, $Leb(aS + b) = 0 \Rightarrow \mu_{x_j}(aS + b) = 0$, but $Leb(S) = 0 \Leftrightarrow Leb(aS + b) = 0$ [55], hence: $Leb(S) = 0 \Rightarrow Leb(aS + b) = 0 \Rightarrow \mu_{x_j}(aS + b) = 0$. Lastly by the semantic definition of $x_i$, we have that $\mu_{x_j}(aS + b) = 0 \Rightarrow \mu_{x_i}(S) = 0$, hence $Leb(S) = 0 \Rightarrow \mu_{x_i}(S) = 0$ by transitivity. All other marginals are unchanged, hence A.C. of each is preserved.

*Case 4.* Sequencing, `if` and `while`: Intuitively since the above statements each preserve A.C of each marginal, any sequencing of them should too. Since the sum of two measures that are both A.C. in each marginal is also A.C. in each marginal, `if` statements preserve A.C. of each marginal. For this same reason `while` loops also preserve A.C.

## 5 Synthesis of Continuity Correction Parameters

We now present our procedure for synthesizing optimal continuity correction parameters which covers lines 6 to 15 in Algorithm 1. This can be thought of as a "training" step which fits the continualized model to the original one. It is important to note that this step is agnostic to the observed data (it only fits to the *Model*), hence it need only be done *once* off-line, regardless of how many times we perform inference on new data sets. Furthermore, even if we do not have parameters to synthesize, this step is still useful for catching runtime errors caused by the approximations, so that we can go back and apply safer approximations if necessary.

### 5.1 Optimization Framework

Ideally the posteriors of our approximated program $\mathcal{T}_{\mathcal{P}}^{\beta}[P]$ and the original $P$, should be reasonably close. However a specific posterior is induced by the corresponding data-set, if our optimization objective tries to minimize the statistical distance from $\mathcal{T}_{\mathcal{P}}^{\beta}[P]$

to $P$, we would simply be over-fitting to the data and we would not be able to re-use $\mathcal{T}_{\mathcal{P}}^{\beta}[P]$ for new data sets with different true parameters. Instead our objective is to minimize the distance between the original *model* $M$, which is simply the fragment of $P$ that does not contain the data or observe block (and hence only defines the prior, likelihood and latent variables), and the corresponding continualized approximation, $\mathcal{T}_{\mathcal{P}}^{\beta}[M]$. To do so, we need to choose the best possible continuity correction factors, $\theta$, for $\mathcal{T}_{\mathcal{P}}^{\beta}[M]$. Thus we define the "optimal" parameters as those which minimize a distance metric $d$ between probability measures $d : \mathbb{M}(\mathbb{R}^n) \times \mathbb{M}(\mathbb{R}^n) \to [0, \infty)$. We also need to ensure that the metric can (a) compute the distance between discrete and continuous distributions and (b) is such that if models or likelihoods are close with respect to $d$, the posteriors should be as well.

**Wasserstein Distance** We choose to use the Wasserstein distance primarily because (1) it can measure the distance between a continuous and discrete distribution (unlike KL-Divergence or Total Variation Distance) and (2) prior work has shown that when performing inference, if using the Wasserstein distance as the chosen metric to approximate a likelihood, the (approximate) posteriors induced are comparable to the true posteriors (obtainable if one used the true likelihood) [49]. Additionally, unlike other metrics, the Wasserstein metric incorporates the underlying difference in geometry of the distributions (which strongly affects inference accuracy [37, 59]).

Let $[\![M]\!](\mu_0)$ represent the *renormalized* measure associated to the observed variables of the original model and let $[\![\mathcal{T}_{\mathcal{P}}^{\beta}[M_\theta]]\!](\mu_0)$ represent the observed variables of the continualized model, but where a given continuity correction factor $\theta$ has been substituted in (both measures start in initial distribution $\mu_0$). Furthermore, let $\mathbf{J} \subseteq \mathbb{M}(\mathbb{R}^2)$ represent the set of all joint measures with marginal measures $[\![M]\!](\mu_0)$ and $[\![\mathcal{T}_{\mathcal{P}}^{\beta}[M_\theta]]\!](\mu_0)$. Hence we now define the 1-Wasserstein Distance:

$$W([\![M]\!](\mu_0), [\![\mathcal{T}_{\mathcal{P}}^{\beta}[M_\theta]]\!](\mu_0)) = \underset{J \in \mathbf{J}}{inf} \int ||x - y|| dJ(x, y) \tag{1}$$

We also provide further justification why the Wasserstein Distance is a sensible metric to use. It is well known that a mixture of Gaussians can converge in distribution to any continuous random variable, however existing work has shown that a mixture of Gaussians can approximate any *discrete* distribution *in the Wasserstein Distance* arbitrarily well [20].

**Objective Function** We now formulate our optimization approach as follows, where $\hat{\theta}$ is the parameter vector minimizing the Wasserstein Distance with respect to the original model $M$, and $d$ is the number of parameters to synthesize.

$$\hat{\theta} = \underset{\theta \in (0,1)^d}{argmin}\ W([\![M]\!](\mu_0), [\![\mathcal{T}_{\mathcal{P}}^{\beta}[M_\theta]]\!](\mu_0)) \tag{2}$$

To restrict the search space we follow common practice [23, 3] by requiring each $\theta_i \in (0, 1)$. Such optimization problem lacks a closed form solution. Symbolically computing the Wasserstein Distance is intractable, hence we numerically approximate it via the empirical Wasserstein Distance (EWD) between observed samples of $M$ and $\mathcal{T}_{\mathcal{P}}^{\beta}[M_\theta]$. Because this step is fully dynamic (we run and sample the model), the samples are conditioned upon successfully terminating, and hence the model's sub-measure has been implicitly *renormalized* to a full probability measure, thus justifying the use of a fully renormalized measure in equations (1) and (2).

**Algorithm 2:** Synthesizing Optimal Continuity Correction Parameters

1 Function Synthesize $P, \mathcal{T}_{\mathcal{P}}^{\beta}[P]$;
**Input :** A program $P$ and a continualized sketch $\mathcal{T}_{\mathcal{P}}^{\beta}[P]$ with $d$ parameters to be synthesized
**Output:** A fully continuous probabilistic program $P_C$ and a binary flag denoting the existence of a runtime error
2 **if** *d==0* **then**
3     $s \leftarrow$`sample`$(\mathcal{T}_{\mathcal{P}}^{\beta}[P], n)$;
4     **if** *s==Error* **then**
5         **return** $\mathcal{T}_{\mathcal{P}}^{\beta}[P]$, *false*
6     **end**
7 **end**
8 **else**
9     $M, \mathcal{T}_{\mathcal{P}}^{\beta}[M] \leftarrow$`getModel`$(P, \mathcal{T}_{\mathcal{P}}^{\beta}[P])$;
10     **for** $\theta_i \in Grid([0,1]^d)$ **do**
11         $p, s \leftarrow$ `Nelder-Mead`$(W, \theta_i, M, \mathcal{T}_{\mathcal{P}}^{\beta}[M], \eta, \epsilon, n)$;
12         **if** *s==Error* **then**
13             **return** $\mathcal{T}_{\mathcal{P}}^{\beta}[P]$, *false*
14         **end**
15         **if** $W(p) < W(\hat{\theta})$ **then**
16             $\hat{\theta} \leftarrow p$
17         **end**
18     **end**
19 **end**
20 **return** `substitute`$(\mathcal{T}_{\mathcal{P}}^{\beta}[P], \hat{\theta})$, *true*

Though intuitively we would expect that as we apply less smoothing (i.e. $\beta < 1$), the optimal $\theta_i$ should also be smaller (less need for correction) and the continualized program should become closer to the original, a simple negative result illustrates this is not always the case and that the dependence between the smoothing and continuity correction *must be* non-linear.

*Remark 1.* $\hat{\theta}$ **cannot be linearly proportional** to $\beta$.

*Proof.* Let $X$ be the constant random variable that is 0 with probability 1 and let $X' \sim Gaussian(0, \beta)$. Furthermore, let $I := (X == 0)$ and $I_c := (c\beta \leq X' \leq c\beta)$ be two indicator random variables. Intuitively we want $I_c$ to have the same probability of being true as $I$ for *any* $\beta$. However if $c$ is constant (such as 1) then $Pr(c\beta \leq X' \leq c\beta)$ will **always** be the same regardless of $\beta$ (when $c = 1$, the probability is always 0.68).

#### 5.2 Optimization Algorithm

Algorithm 2 presents our approximate synthesis algorithm, which is called as a subroutine in the main algorithm. As seen in line 2, if there are no parameters to be synthesized ($d == 0$) we still sample the continualized program in hopes of uncovering a possible runtime error (or gaining statistical confidence that one does not occur). We check for such an error in line 4 and if one exists, we return immediately, with the flag variable set to *false* (line 5).

To evaluate the EWD objective function (when there are parameters to synthesize), Algorithm 2 follows a technique from [14] and uses a Nelder-Mead search (line 11), due to Nelder-Mead's well known success in solving non-convex program synthesis problems. We first extract the fragment of the programs corresponding to the models, $M$ and $\mathcal{T}_{\mathcal{P}}^{\beta}[M]$, respectively in line 9. In each step of the Nelder-Mead search we take $n$ samples ($n \approx 500$) of $\mathcal{T}_{\mathcal{P}}^{\beta}[M]$, but with a fixed value of $\theta_i$ substituted into $\mathcal{T}_{\mathcal{P}}^{\beta}[M]$, to compute the EWD with respect to samples of the original model $M$ (which have been cached to avoid redundant resampling). The Nelder-Mead search steps through the parameter space (with step size $\eta > 0$), substituting different values of $\theta$ into $\mathcal{T}_{\mathcal{P}}^{\beta}[M]$. This process continues until the search converges to a minimizing parameter, $p$, that is within the stopping threshold $\epsilon > 0$ or encounters a runtime error during the sampling (which is checked in line 12). As before, if we encounter such an error we immediately return with the flag set to false (line 13). Following [14], we successively restart the Nelder-Mead search from $k$ evenly spaced grid points in $[0,1]^d$ (hence the loop in line 10), to find the globally optimal parameter (hence our approach is robust to local minima), which we successively update in lines 15-16. If no runtime error was ever encountered, we substitute in the parameters with the minimum EWD over all runs, $\hat{\theta}$, to the fully continuous program $\mathcal{T}_{\mathcal{P}}^{\beta}[P]$ and return (line 20). Though it can be argued this sampling is potentially as difficult as the original inference, we reiterate that we need only do this once offline, hence the cost is easily *amortized*.

## 6 Methodology

### 6.1 Benchmarks

Table 1 presents the benchmarks. For each benchmark, Columns 2 and 3 present the original prior and likelihood type, respectively. Column 4 presents whether the continuity correction was applied. Column 5 presents the time to continualize the program, $T_{Cont.}$. As can be seen in Columns 4 and 5 the *total* continualization time, $T_{Cont.}$, depends on whether parameters had to be synthesized. `GPAExample` had the longest $T_{Cont.}$ at $3.6s$, due to the complexity of the multiple predicates, however these times are *amortized* as our synthesis step is done only once.

As our problem has received little attention, no standard benchmark suites exist. In fact, to make inference tractable, for many models, developers would construct continuous approximations by hand, in an ad hoc fashion. However we wanted a benchmark suite that showcased all 3 inference scenarios that our approach works for: (1) discrete/hybrid prior and discrete/hybrid likelihood (2) continuous prior but discrete/hybrid likelihood and (3) discrete/hybrid prior but a continuous likelihood. Therefore, we obtained the benchmarks in two ways. First, we looked at variations of the mixed distributions benchmarks previously published in the machine learning community, e.g., [65, 58], which served as the inspiration for our `GPAExample`. Second, we took existing benchmarks [27, 30] for which designers modeled certain distributions with continuous approximations, and we retro-fitted these models with the corresponding discrete distributions. This step was done for `Election, Fairness, SVMfairness, SVE`, and `TrueSkill`. These discretizations were only applied where they made sense, e.g., the `Gauss(np,np(1-p))` in the original Election program became discretized as `Binomial(n,p)`. We also took popular Bayesian models from Cognitive Science literature which use multiple discrete latent variables [39] and these models

Table 1: Description of Benchmarks

| Program | Prior | Likelihood | Correction? | $T_{Cont.}$ (s) |
|---|---|---|---|---|
| GPAExample | Uniform | Discrete | ✓ | 3.643 |
| Election [27] | DiscUniform | Bernoulli | ✓ | 1.139 |
| Fairness [2] | DiscUniform | Bernoulli | ✓ | 1.809 |
| SVMfairness [2] | Binomial | Continuous | ✓ | 1.578 |
| TrueSkill [30] | Poisson | Bernoulli | ✓ | 1.149 |
| DiscreteDisease | DiscUniform | Discrete | × | 0.006 |
| SVE [58] | Uniform | Hybrid | × | 0.009 |
| BetaBinomial [39] | Beta | Discrete | × | 0.006 |
| Exam [39] | Uniform | Discrete | × | 0.008 |
| Plankton [10] | DiscUniform | Discrete | × | 0.006 |

are `BetaBinomial` and `Exam`. Lastly we took population models from the mathematical biology literature [10, 4] to build benchmarks since populations are by nature discrete. This was done for `Plankton` and `DiscreteDisease`. We present the original programs in the appendix [38].

**Implementation** We implemented Leios in Python (~4.5K LoC). All experiments were run on an Intel Xeon, multi-core desktop running Ubuntu 16.04 with a 3.7 GHz CPU and with 32GB RAM. All results are obtained from single-core executions.

### 6.2 Experimental Setup

**Continualized Versions** As there are no other general tools that automatically continualize probabilistic programs in mainstream languages, we compare Leios with:

- ***Original Program***: inference done in standard fashion on the original model, and
- ***Naive Smoothing***: inference done on a KDE style model in which Gaussian smoothing is applied *only* to the observed variable, but no approximations are applied to the inner latent variables.

We will refer to these as simply "Original" and "Naive" respectively.

**Inference Accuracy Comparison using Ground Truth** Our experimental design compares the respective inference estimates with the *ground truth*. We set the experiments as follows: For each of the original discrete or hybrid programs $P$, we replace the program variable corresponding to the prior distribution with a fixed value $\tau$ (the ground-truth) to obtain $P(\tau)$. We then sample $P(\tau)$ to obtain 25 observed data points, which will be used to test inference performance on $P$, $P_{NS}$, and $P_{Leios}$ respectively. To test inference performance we then score $P$ (original program), $P_{NS}$ (naively smoothed program), and $P_{Leios}$ against the observed data points to infer the posterior over the ground truth parameter $\tau$. Note the programs only have access to the data samples, but not $\tau$.

For each of the 3 versions: $P$, $P_{NS}$, and $P_{Leios}$, we take the inferred posterior means as the estimates of the value, and then compare it with the ground-truth value $\tau$ to measure the error ratio $E = \left|\frac{\tau - \tau_{est}}{\tau}\right|$. This entire procedure is repeated for 10 different values of $\tau$ to get a representative average of inference performance over a wide range of true parameter values.

Table 2: Inference Times (s) and Error Ratios for each model, $\beta = 0.1$

| Program | Original Time | Original Error | Naive Time | Naive Error | Leios Time | Leios Error |
|---|---|---|---|---|---|---|
| GPAExample | 0.806 | 0.090 | 0.631 | 0.070 | 0.605 | 0.058 |
| Election | - | - | 3.232 | 0.051 | 0.616 | 0.036 |
| Fairness | 4.396 | 0.057 | 0.563 | 0.056 | 0.603 | 0.093 |
| SVMfairness | - | - | 0.626 | 0.454 | 0.980 | 0.261 |
| TrueSkill | 3.668 | 0.009 | 0.494 | 0.059 | 0.586 | 0.053 |
| DiscreteDisease | 4.944 | 0.009 | 1.350 | 0.013 | 0.490 | 0.008 |
| SVE | - | - | 0.522 | 0.045 | 0.516 | 0.091 |
| BetaBinomial | 1.224 | 0.028 | 0.564 | 0.024 | 0.459 | 0.013 |
| Exam | 3.973 | 0.087 | 0.504 | 0.126 | 0.527 | 0.133 |
| Plankton | 0.570 | 0.017 | 0.457 | 0.080 | 0.453 | 0.042 |
| Average | 2.797 | 0.043 | 0.894 | 0.098 | 0.584 | 0.079 |

**Analyzed Probabilistic Programming Systems.** We used two languages in our development: WebPPL [26] (with MCMC inference) and Pyro [8] (with Variational inference). Our implementation automatically generates WebPPL code for all the programs. We used 3500 MCMC samples (with burn-in of 700 samples) in the simulation. For Pyro, we only wanted to test *fully-automatic* black-box Variational Inference, hence we did not manually marginalize out discrete variables (which is often not even applicable, as the discrete variables are the one we wish to estimate).

**Inference Time Measurement** We measure the time taken for inference for each version using built-in timers (which exclude file reading and warm-up). A timeout of 10 minutes was used for the inference step. We used this same procedure for both MCMC-based sampling in WebPPL and Variational Inference in Pyro.

### 7 Evaluation

We study the following three research questions:

**RQ1** Can program continualization make inference faster, while still maintaining a high degree of accuracy, compared to the original program and naive smoothing?

**RQ2** How do performance and accuracy vary for different smoothing factors $\beta$?

**RQ3** Can program continualization enable running transformed programs with off-the-shelf inference algorithms that cannot execute the original programs?

#### 7.1 RQ1: Benefits of Continualization

Table 2 presents detailed timing and accuracy errors for a single smoothing factor $\beta$ on WebPPL programs. Columns 2 and 3 present the time and error (compared to the ground truth) for the original program. Columns 4 and 5 present time/error for the naive smoothing and Columns 6 and 7 present time/error for Leios.

From Table 2 we can see that on average, Leios leads to faster inference than both the Original (no approximations) and Naive (0.584s vs 2.797s and 0.894s, respectively). The Naive version was also faster than the original, giving more evidence that continuous models (even when just the observed variable is continualized) yield faster inference.

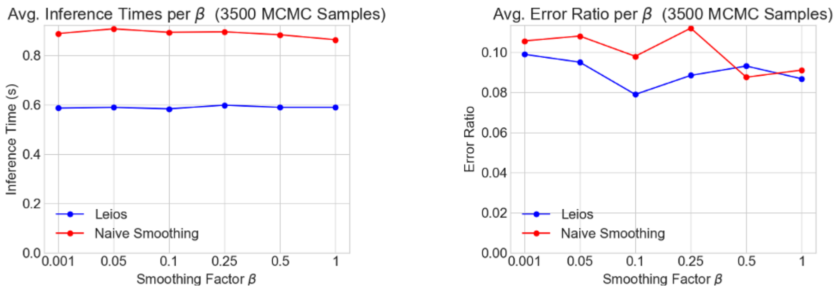

(a) Avg. Inference Time (b) Avg. Error Ratio

Fig. 7: Inference Times and Error ratios for Leios and Naive for different $\beta$

For accuracy, inference performed via Leios was on average more accurate than Naive ($E = 0.079$ vs. 0.098, respectively). Both were slightly less accurate than inference performed on Original ($E = 0.043$). This is not unreasonable as Original has *no* approximations applied (which are the main source of inference error). However the Original failed on `Election`, `SVE`, and `SVMfairness`. For `Election`, a large Binomial latent led to a timeout, and it also slowed the Naive version relative to Leios (3.23s vs 0.61s). The Original failed on `SVE` since it is a hybrid discrete-continuous model (which can make inference intractable [65, 6]). `SVMfairness` is a non-linear model where many latent variables have high variances, leading to inference on the Original failing to converge; Leios and Naive had higher error on this benchmark, for much the same reason (though Leios was still significantly better than Naive, $E = 0.261$ vs 0.454).

Although Leios was faster than Original in all cases, for `TrueSkill` and `SVMfairness`, Leios was somewhat slower than Naive. This is likely because the discrete latent variables in these benchmarks had small enough parameters (Binomial with small $n$). Similarly, for `Fairness`, Leios was slightly less accurate than Naive because the Gaussian approximation can be less accurate for smaller $n$.

#### 7.2 RQ2: Impact of Smoothing Factors

Figure 7 presents the average inference times and ERs for different smoothing factors $\beta$. In both cases, X-axes represent smoothing factors. The Y-Axis of the left subfigure presents time, and Y-Axis of the right presents error ratio compared to the ground truth (less is better).

Figure 7 (a) shows that Inference on the programs constructed by Leios is non-trivially faster than inference done on the naively smoothed version, regardless of the $\beta$ used (which has negligible affect on the inference time for the $\beta$ we examined).

Figure 7 (b) presents how accuracy directly depends on $\beta$. The Error Ratio for Leios reaches a local minimum when $\beta = 0.1$. Because Leios achieves "global" smoothing by approximating each latent, a larger value for $\beta$ is not needed (unlike Naive). We also noticed for many benchmarks, smaller $\beta$ led to better continuity correction parameters which also leads to better inference. Naive's performance suffers for smaller $\beta$, which we attribute to small $\beta$ creating a highly multimodal observed variable distribution (also presented in Section 2) which hampers inference [37, 59]. Consequently, Naive performs best when $\beta = 0.5$, however this $\beta$ introduces non-trivially higher variance, which may often negatively affect the precision of inference.

Table 3: Variational Inference Times (s) and Error Ratios for selected $\beta$

| | | | | | $\beta: 0.25$ | | $\beta: 0.5$ | | $\beta: 0.75$ | |
|---|---|---|---|---|---|---|---|---|---|---|
| Program | $T_{org}$ | $E_{org}$ | $T_{NS}$ | $E_{NS}$ | $T_{Leios}$ | $E_{Leios}$ | $T_{Leios}$ | $E_{Leios}$ | $T_{Leios}$ | $E_{Leios}$ |
| GPAExample | - | - | - | - | 3.111 | 0.207 | 3.341 | 0.241 | 3.435 | 0.321 |
| Election | - | - | - | - | 1.762 | 0.070 | 1.755 | 0.110 | 1.764 | 0.064 |
| Fairness | - | - | - | - | 1.813 | 0.722 | 1.827 | 0.769 | 1.830 | 0.753 |
| SVMfairness | - | - | - | - | 1.800 | 0.201 | 1.806 | 0.293 | 1.804 | 0.301 |
| TrueSkill | - | - | - | - | 1.809 | 0.119 | 1.802 | 0.062 | 1.790 | 0.090 |
| DiscreteDisease | - | - | - | - | 1.734 | 0.248 | 1.731 | 0.471 | 1.747 | 0.553 |
| SVE | 0.677 | 0.684 | 1.478 | 3.095 | 1.471 | 0.587 | 1.460 | 0.566 | 1.448 | 0.348 |
| BetaBinomial | - | - | - | - | 1.605 | 0.834 | 1.596 | 0.708 | 1.587 | 0.497 |
| Exam | - | - | - | - | 0.603 | 0.222 | 0.602 | 0.213 | 0.603 | 0.285 |
| Plankton | - | - | - | - | 3.432 | 0.297 | 3.427 | 0.763 | 3.434 | 0.530 |

#### 7.3 RQ3: Extending Results to Other Systems

Table 3 presents the results for running translated programs in Pyro. Columns 2-5 present the inference times and result errors for the original and naively smoothed program. These columns are "-" when Pyro cannot successfully perform inference (i.e. the model contains a discrete variable that is unsupported by the auto guide). Columns 6-11 present Leios' time and error for each model, for three different smoothing parameters.

Fully-automated Variational Inference failed on all but one of the examples for both the Original and Naive. This is because in both cases the program still contains latent or observed discrete random variables. For most of the benchmarks (`Election`, `GPA`, `TrueSkill`) the program optimized with Leios had errors comparable to those computed previously with MCMC in WebPPL. For some the error was over 0.5 for all $\beta$ (`BetaBinomial`, `Fairness`), which is in part a consequence of limitations of automatic VI, and hence for certain models manual fine-tuning may be unavoidable. These results illustrate that Leios can be used to create an efficient program in situations when the original language does not easily support non-continuous distributions.

### 8 Related Work

**Probabilistic Program Synthesis** To the best of our knowledge, we are the first to study program transformations that approximate discrete or hybrid discrete-continuous *probabilistic programs* with fully continuous ones to improve inference. Probabilistic program synthesis takes a more ambitious task of generating probabilistic programs with certain properties directly from data. For instance, Nori et al. [51] aim to synthesize a probabilistic program given a program sketch and a data-set to fit the program to. However, it merely fits the distribution parameters to the sketch. Furthermore their language lacks '==' comparisons. Chasins et al. [11] takes a similar approach but only apply continuous approximations to *already* continuous variables.

**Probabilistic Inference with Discrete and Hybrid Distributions** Recent work [65, 66] has explored developing languages and semantics to encode discrete-continuous mixtures, however these all restrict the types of programs that can be expressed and require specialized inference algorithms. In contrast, Leios can work with a variety of off-the-shelf inference algorithms that operate on arbitrary models and does not need to define its own inference algorithm. In [66] the authors explored a restricted programming language that can statically detect which parameters the program's density is discontinuous in. However they did not address the question of continuous approximation, rather their approach was to develop a custom inference scheme and restrict the language so that pathological models cannot be written (they also disallow '==' predicates). In [65], Wu et al. develop a custom inference method for discrete-continuous mixtures but only for models encodeable as a Bayesian network, furthermore as pointed out by [47], the specialized inference method of Wu et al. is restrictive since it cannot be composed with other program transformations.

Additionally, Machine Learning researchers have developed other continuous relaxation techniques to address the inherent problems of non-differentiable models. One other popular method is to reparametrize the gradient estimator during Variational Inference (VI) computation, commonly called the "reparameterization trick" [42, 61]. However, this approach suffers from the fact that not all distributions support such gradient reparameterizations, and also this method is only limited to Variational Inference. Conversely our approach allows one to still use *any* inference scheme. Further, even though these techniques have been attempted in the probabilistic programming setting, [40], such work still inherits the aforementioned weaknesses.

We also draw upon Kernel Density Estimation (KDE) [62], a common approximation scheme in statistics. KDE fits a Kernel density to each observed data point, hence constructing a smooth approximation. Naive Smoothing is essentially a KDE (with a Gaussian Kernel) of the original while Leios employs additional continualizations. Furthermore, our smoothing factor $\beta$ is analogous to the *bandwidth* of a KDE.

**Program Analysis for Probabilistic Programs** Multiple Program Analysis frameworks and systems have been developed for Probabilistic Programming [57, 33, 63, 32, 22]. Additionally these analyses make use of a rich set of semantics [44, 36, 7, 64, 19], however of particular note is recent work by Lew et al. [41], which provides a type system for reasoning about variational approximations; however they focus on continuous approximations of already continuous variables.

**Benefits of Continuity in Conventional Programs** The idea of smoothing and working with continuous functions in non-probabilistic programs has found success in a variety of applications [21, 12, 34, 13]. Our work derives inspiration mainly from Smooth interpretation [14], which provides a semantics for smoothing *deterministic* programs encoding a discontinuous or discrete function.

### 9 Conclusion

We presented Leios as a method for approximating probabilistic programs with fully continuous versions. Our approach shows that by continualizing probabilistic programs, it is possible to achieve substantial speed-ups in inference performance whilst still preserving a high degree of accuracy. To this effect we combined two key techniques: statement level program transformations to continualize latent variables and a novel continuity correction synthesis procedure to correct branch conditions.

**Acknowledgements**

We would like to thank the anonymous reviewers for their constructive feedback. We thank Darko Marinov for his helpful feedback during early stages of the work. We thank Adithya Murali for valuable feedback about the semantics. We thank Zixin Huang and Saikat Dutta for helpful discussions about the evaluation and Vimuth Fernando and Keyur Joshi for helpful proofreads. JL is grateful for support from the Alfred P. Sloan foundation for a Sloan Scholar award used to support much of this work. The research presented in this paper has been supported in part by NSF, Grant no. CCF-1846354.
